# Differentiation of neuropsychological features between posterior cortical atrophy and early onset Alzheimer’s disease

**DOI:** 10.1186/s12883-018-1068-6

**Published:** 2018-05-10

**Authors:** Jieying Li, Liyong Wu, Yi Tang, Aihong Zhou, Fen Wang, Yi Xing, Jianping Jia

**Affiliations:** 1 Innovation Center for Neurological Disorders, Department of Neurology, Xuan Wu Hospital, Capital Medical University, Beijing, People’s Republic of China; 2 Beijing Key Laboratory of Geriatric Cognitive Disorders, Beijing, People’s Republic of China; 3 Clinical Center for Neurodegenerative Disease and Memory Impairment, Capital Medical University, Beijing, People’s Republic of China; 4 Center of Alzheimer’s Disease, Beijing Institute for Brain Disorders, Beijing, People’s Republic of China; 5 Key Laboratory of Neurodegenerative Diseases, Ministry of Education, Beijing, People’s Republic of China; 6 National Clinical Research Center for Geriatric Disorders, Beijing, People’s Republic of China; 7 The Second People’s Hospital of Guiyang, Guizhou, People’s Republic of China

**Keywords:** Posterior cortical atrophy, Early onset Alzheimer’s disease, Cognition, Neuropsychology

## Abstract

**Background:**

Posterior cortical atrophy (PCA) is a group of clinical syndromes characterized by visuospatial and visuoperceptual impairment, with memory relatively preserved. Although PCA is pathologically almost identical to Alzheimer’s disease (AD), they have different cognitive features. Those differences have only rarely been reported in any Chinese population. The purpose of the study is to establish neuropsychological tests that distinguish the clinical features of PCA from early onset AD (EOAD).

**Methods:**

Twenty-one PCA patients, 20 EOAD patients, and 20 healthy controls participated in this study. Patients had disease duration of ≤4 years. All participants completed a series of neuropsychological tests to evaluate their visuospatial, visuoperceptual, visuo-constructive, language, executive function, memory, calculation, writing, and reading abilities. The cognitive features of PCA and EOAD were compared.

**Results:**

All the neuropsychological test scores showed that both the PCA and EOAD patients were significantly more impaired than people in the control group. However, PCA patients were significantly more impaired than EOAD patients in visuospatial, visuoperceptual, and visuo-constructive function, as well as in handwriting, and reading Chinese characters.

**Conclusions:**

The profile of neuropsychological test results highlights cognitive features that differ between PCA and EOAD. One surprising result is that the two syndromes could be distinguished by patients’ ability to read and write Chinese characters. Tests based on these characteristics could therefore form a brief PCA neuropsychological examination that would improve the diagnosis of PCA.

## Background

Posterior cortical atrophy (PCA) is manifests mainly as early visual dysfunction in the context of neurodegeneration of posterior cortical regions. Benson [1] first described this syndrome and proposed that it be independently classified. The main clinical features, besides space perception (visuospatial) and object perception (visuoperceptual) deficits, include alexia, Balint syndrome (simultanagnosia, ocular apraxia, and visual ataxia), Gerstmann’s syndrome (acalculia, agraphia, digital agnosia, and left and right confusion), with relative preservation of memory and insight [2]. PCA usually occurs from the mid-50s to early 60s and may account for 5 to 10% of cases diagnosed as early-onset Alzheimer’s disease (EOAD) [3].

Neuropathological studies have indicated demonstrated that most cases of PCA are identical to Alzheimer’s disease [4–6]. In some rare instances, corticobasal degeneration, Lewy bodies dementia and prion disease cause PCA [4, 5]. In cases of Alzheimer’s disease pathology, PCA has also been recognized and described in consensus criteria for atypical Alzheimer’s disease [7], as well as a “visual variant of Alzheimer’s disease” [8]. For a clearer understanding of the pathogenesis in AD, it is crucial to distinguish the cognitive features of PCA from those of AD. Moreover, with the multiple pathologies that underlie the PCA syndrome [5], pharmacological treatment and behavioral interventions for PCA may differ from those of AD.

Several studies have evaluated clinical differences between PCA and AD, listing core clinical and cognitive features for PCA [4, 6, 9–11]. PCA also has significant features in the form of visuospatial and visuoperceptual symptoms. However, there are some inconsistencies exist among the cognitive features described in previous studies. Mendes et al. [6] found that verbal fluency was untouched by PCA, but Tang-wai et al. [4] did not. Charles and Hillis [9] and McMonagle et al. [10] found the visual constructional ability of PCA patients was significantly more impaired than in typical AD, which contradicted findings by Mendes. McMonagle et al. [10] and Kas et al. [11], unlike other research teams, found working memory to be more prominently impaired in PCA than in AD.

The origins of the discrepancies in these findings may lie in the use of different neuropsychological tests, different research contexts, different populations, or differences in the duration of disease. Past studies have generally not included information regarding the pathology underlying PCA. This has had several consequences. First, clear differentiation of PCA from EOAD might benefit from the application of a broader set of neuropsychological measures. Second, verification of pathology is necessary to validate neuropsychological differences. That verification is particularly important, because cognitive testing may be more readily available and less expensive than other diagnostic techniques (e.g., PET imaging). The use of these neuropsychological indicators may be efficacious in the diagnosis of PCA, particularly early diagnosis.

The five studies that we found were all performed on Euro-American participants, and it seems feasible that cultural differences may affect the manifestations of PCA. China, with its own cultural characteristics, has a population estimated at more than 1.4 billion people, a substantial proportion of whom fall into the age group most likely to be affected by PCA and EOAD. For this reason, it is important, as both a practical medical concern and a potentially valuable theoretical perspective, to examine neuropsychological factors in Chinese PCA and EOAD patients. We further note that many Chinese people may have limited access to or limited ability to afford sophisticated diagnostic techniques (e.g., imaging), so a quick preliminary screening process would be beneficial.

For this reason, we compared cognitive manifestations of PCA and EOAD in Chinese patients during early stages of the disease. We used a series of neuropsychological tests to identify cognitive features of PCA that distinguish it from EOAD.

## Methods

### Participants

We recruited patients from the Department of Neurology at Xuan Wu Hospital, Capital Medical University, Beijing, from 2013 to 2016. Healthy controls came from the physical examination center at the same hospital. The clinical sample included 21 PCA and 20 EOAD patients. Patients with PCA met the standard clinical criteria for PCA developed by the AAIC International Working Group [12]. Core clinical features included insidious onset and gradual progression; prominent visuoperceptual and visuospatial impairments without significant impairment of vision itself; evidence of complex visual disorders (e.g., elements of Balint’s syndrome or Gerstmann’s syndrome, visual field defects, visual agnosia, environmental disorientation); relative preservation of memory and insight; and absence of stroke or tumor. Supportive features included presenile onset, alexia, ideomotor, or dressing apraxia, prosopagnosia, and prolonged color after-images. Structural neuroimaging with either MRI or CT showed pronounced atrophy of the parietotemporo-occipital cortex, and ^18^F-fluoro-deoxyglucose (FDG) PET or single photon emission computed tomography (SPECT) showed hypometabolism or hypoperfusion in the occipital and parietal lobes. We excluded patients who had a clear history of vascular disease, severe white matter lesions, Lewy body dementia, corticobasal degeneration, frontotemporal dementia, primary progressive aphasia, hydrocephalus, pre-morbid dyslexia or dysgraphia, or other acute neurological diseases. We also excluded patients with non-AD pathology by brain amyloid imaging. All patients were clinically followed up for at least 12 months to support the diagnosis of PCA.

Patients with EOAD met the IWG-2 diagnostic criteria [7] and exhibited symptom onset prior to age 65. We excluded patients with a history of stroke, traumatic brain injury, chronic alcoholism, or vascular dementia. We recruited the patients who had PCA and EOAD for ≤4 years. We defined disease duration as the time from the onset of the first symptoms observed by the patients themselves or by caregivers at the time the medical records started.

We recruited 20 healthy controls from the physical examination center of Xuan Wu Hospital, Capital Medical University. Participants met the following criteria: no memory complaints; cognitively normal, based on the absence of significant impairment in cognitive functions or activities of daily living (ADL); Clinical Dementia Rating (CDR) = 0; and Mini-Mental State Examination (MMSE) > 26; they had sufficient visual and auditory acuity to allow cognitive testing. Participants with any significant neurologic disease, psychiatric disorders, or psychotic features were excluded.

All the patients completed imaging including brain MRI or CT, and FDG-PET or SPECT. Twelve PCA patients and 20 EOAD patients completed [^11^C]-labeled Pittsburgh compound B (PIB) PET. The method of data processing was described in previous study [13]. Positive PIB PET results were recorded for 9 of 12 PCA cases and all EOAD patients and negative results indicative of non-AD pathology in 3 of the 12 PCA patients. We excluded 3 non-AD pathology PCA cases. Nine PCA patients refused to undergo PIB PET due to the high cost of PET (approximately US$ 900, and not covered by insurance). However, we followed up those patients for at least 12 months to exclude Lewy body dementia, corticobasal degeneration, and prion disease; consequently, even in the absence of the PET scan, we were confident of the PCA diagnosis. This study was approved by the Ethics Committee of Xuan Wu Hospital of Capital Medical University. We obtained written informed consent from each participant or caregiver.

### Neuropsychological tests

All participants completed a battery of neuropsychological assessment measures, including the following: (1) Global cognitive function was assessed using several tests: the Mini-Mental State Examination (MMSE) [14]; the Montreal Cognitive Assessment (MoCA) [15]; and the Clinical Dementia Rating (CDR) [16]. The MMSE cutoff value for normal Chinese populations is ≥26, with education ≤12 years. The MoCA cutoff value for normal Chinese populations is ≥24, with education ≤12 years. (2) Episodic memory was assessed using the World Health Organization University of California-Los Angeles Auditory Verbal Learning Test (AVLT) [17], including the AVLT-immediate, AVLT-delay, AVLT-clue, and AVLT- recognized. The cutoff score for immediate recall is ≥18; the delayed recall cutoff score is ≥6. (4) Language was assessed using the Boston Naming Test, BNT [18], and verbal fluency (naming as many animals as possible in 1 min). (5) Visuo-constructive function was assessed using the Rey-Osterrich complex figures test of direct copying [19] (using 36-point scoring). (6) Attention and working memory were assessed using forward and backward digit span subtests from Wechsler Adult Intelligence Scale [20]. (7) Executive function was assessed using the Trail Making Test Parts A and B [21]. (8) Mental calculation was assessed using the calculation subtest from the MMSE (Series of Seven Subtraction Test, SSST), and unit conversion (e.g., how many Fens (cents) in one Yuan (dollar)). (9) Writing was assessed by spontaneous handwriting and copying a short sentence from the Aphasia Battery of Chinese [22]. Performance was scored by number of words written correctly. (10) Reading was assessed by reading a short sentence, and six lines of text (a total of 118 individual words) selected from a news report. Words were in black Songti font, size 16, presented on a white background. Participants were given a maximum of 300 s to read each passage. For each word, participants who took more than 10 s to respond were prompted to move onto the next word. Participants were not discouraged from using their finger to maintain their place when reading. We used the method developed by Yong KX et al. [23, 24]. Performance was scored by number of words correctly read, regardless of word order. Lines skipped and word misread or repeated were also recorded. (11) Visual-spatial function was assessed by tests of bell cancellation [25] (total number of omissions is 35; performance was scored as the actual number of bells omitted), the five series of overlapping figure [26] (recognize each single object from four overlapping figure), and Navon figures [27] (12 Navon figures consisting of a large character (the global level) made out of small characters (the local level)). (12) Visual-perceptual functioning was assessed using a matching test (five objects function matching, and five shapes matching), and the Cookie Theft pictures from the Boston Diagnostic Aphasia Examination [28]. PCA patients also underwent the posterior neuropsychological battery, developed by Kas et al. [11]. That battery included assessment of Balint’s syndrome, Gerstmann’s syndrome, visual neglect, visual agnosia, limb apraxia, and agraphia.

All participants underwent the neuropsychological assessment on the day of the MRI scan and all were evaluated by the same neuropsychologist, who was blind to the participants’ diagnoses. The battery of neuropsychological tests took 2–3 h. PCA patients were given a half hour to rest between the two batteries of tests.

### Statistical analysis

Differences between the groups on the neuropsychological tests scores were assessed with one-way ANOVA followed by pairwise comparisons performed with Bonferroni tests, or Kruskal-Wallis H tests followed by the post-hoc tests with pairwise comparisons for test scores that showed deviations from the non-normal distribution. The statistical analysis software reported *p* values that had been adjusted for the multiple pairwise comparisons between the three groups. The duration of the disease was analyzed using the Mann-Whitney test.

## Results

### Demographic and clinical characteristics

We collected demographic data and medical histories from the patients themselves or from their caregivers. Tables 1 and 2 summarize group demographic and clinical characteristics. There was no significant difference in age, sex, or education among the three groups. Also, there was no significant difference in the disease duration (*p* = .602) between PCA and EOAD patients (Table 1).

**Table 1:** Demographic data for patients diagnosed with posterior cortical atrophy (PCA) and early onset Alzheimer’s disease (EOAD). Controls were healthy adults with no apparent brain pathology

Sample characteristics	PCA	EOAD	Controls	*p*
	(*n* = 18)	(*n* = 20)	(*n* = 20)	
Female/male	10/8	12/8	12/8	.669^a^
Age (SD), years	57.5 (6.1)	52.5 (7.3)	52.5 (7.7)	.054^b^
Disease duration (SD), years	3 (2)	3 (1.8)	–	.602^c^
Education (SD), years	10.2 (3.7)	10.7 (4.5)	12.4 (4.1)	.235^b^
PIB-PET (+/−) (*n* = 9)^d^	9/0	20/0	–	

Note: Age, disease duration, and education did not differ significantly among the three groups

+, positive PIB PET scan; **−**, negative PIB PET scan

^a^Chi-square test

^b^One-way ANOVA

^c^Mann-Whitney test

^d^9 of the PCA patients refused the PIB-PET scan

**Table 2:** Visual characteristics of individual patients diagnosed with posterior cortical atrophy (PCA; *n* = 18)

	Patients Number																	
Symptom	1	2	3	4	5	6	7	8	9	10	11	12	13	14	15	16	17	18
Visual agnosia																		
Object agnosia		+							+	+								
Prosopagnosia						+						+						
Color agnosia			+															
Balint’s syndrome																		
Simultanagnosia	+	+	+	+	+	+	+	+	+	+	+	+	+	+	+		+	
Ocular apraxia						+		+	+	+		+					+	
Optic ataxia		+	+	+	+	+		+	+			+	+				+	
Gerstmann’s syndrome																		
Right–left indistinction		+			+		+	+	+		+		+		+	+		
Agraphia	+	+	+	+	+		+	+	+	+	+	+	+	+	+	+	+	
Finger agnosia		+		+	+	+	+		+	+	+		+		+	+		
Acalculia	+	+		+	+	+	+	+	+	+	+	+	+	+	+	+	+	+
Hemineglect		+	+			+				+	+	+					+	+
Limb apraxia		+		+	+	+	+	+	+	+	+		+	+		+		
Visuo-constructive apraxia	+	+	+	+	+	+	+	+	+	+	+	+	+	+	+	+	+	+
Alexia	+	+	+	+		+	+	+	+	+	+	+	+	+	+	+	+	+

The plus signs indicate the presence of symptoms

### Clinical characteristics of PCA patients

Clinical characteristics of PCA patients were found to be consistent with the core features of PCA (Table 2). Sixteen patients (89%) presented with simultanagnosia. Seventeen patients (84%) had incomplete Gerstmann’s syndrome. All 18 PCA patients presented with visuo-constructive apraxia, and 17 patients (84%) had concurrent varying degrees of alexia. Sixteen patients (89%) showed memory impairment. Other features included object agnosia (17%), prosopagnosia (11%), abnormal color perception (6%), hemineglect (44%), and limb apraxia (67%).

### Comparison of PCA and EOAD on neuropsychological tests

**Fig. 1:**
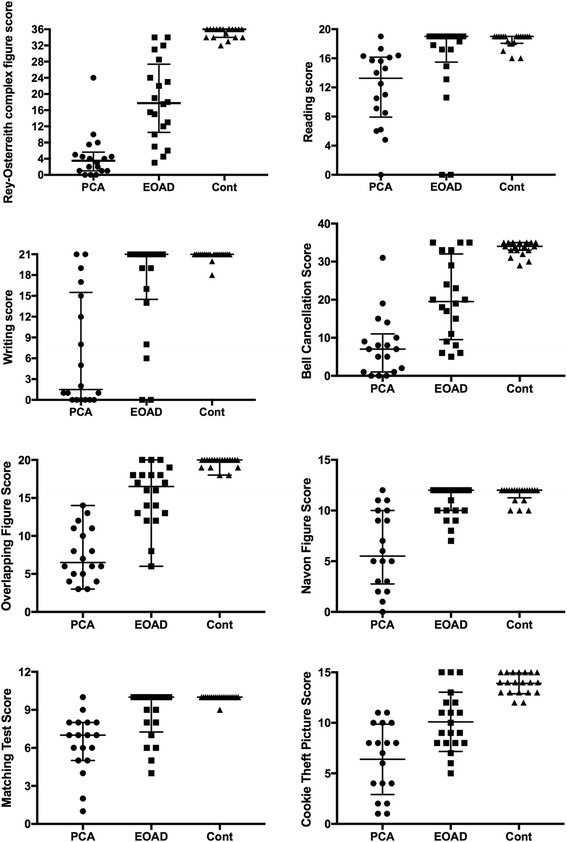
Scatter-plots representing Rey-Osterreith complex figure, writing, reading, bell cancellation, overlapping figure, Navon figure, matching test, and cookie theft picture scores for posterior cortical atrophy (PCA; *n* = 18), early onset Alzheimer’s disease (EOAD; *n* = 20), and healthy controls (Cont; *n* = 20)

Table 3 and Fig. 1 summarize group performances on the neuropsychological tests we administered. Scores on all of the neuropsychological tests showed that both the PCA and EOAD patients were significantly more impaired than in the control group (all *p* < 0.05). The PCA patients exhibited significantly worse performance than EOAD patients in the Rey-Osterrich complex figures tests (*p* = .011). All of PCA patients scored under the cutoff scores in Rey-Osterreith complex figure copy.

**Table 3:** Neuropsychological test scores for samples of patients diagnosed with posterior cortical atrophy (PCA; *n* = 18) and early onset Alzheimer’s disease (EOAD; *n* = 20)

Neuropsychological tests	PCA	EOAD	*P* value
MMSE score (30)	14 (8.8)	13 (9)	> 1.000^a^
MoCA score (30)	9.8 (5.7)	11.3 (5.9)	> 1.000^b^
CDR - SOB (18)	6 (5.1)	6.5 (4.5)	> 1.000^a^
Attention & working memory			
Digit span forward (11)	7 (1.5)	7 (2)	> 1.000^a^
Digit span backward (9)	3 (1)	3 (1)	.898^a^
Executive function			
Trail Making Test A Score (24)	0 (11)	17.5 (24)	.087^a^
Trail Making Test B Score (24)	0 (0)	0 (10.5)	.794^a^
Language			
Boston naming test (30)	13.5 (12.8)	17 (10.5)	.375^a^
Verbal fluency	9.6 (3.6)	8.9 (3.3)	> 1.000^b^
Visuo-constructive function			
Rey-Osterreith complex figure copy (36)	3.5 (4.6)	17.8 (16.9)	.011^a^
Memory			
AVLT-immediate (45)	11.6 (5.3)	11.6 (7.0)	> 1.000^b^
AVLT-delay (15)	0 (2.3)	0 (1)	> 1.000^a^
AVLT-clue (15)	2 (3)	3 (5.5)	> 1.000^a^
AVLT-recognized (15)	2.5 (4.3)	3.5 (5.5)	> 1.000^a^
Calculation (8)	0.5 (2)	2 (3.75)	.141^a^
Writing test (21)	1.5 (15.5)	21 (6.5)	.002^a^
Reading test (19)	13.3 (8.2)	19 (3.5)	.002^a^
Visual-spatial			
Bell cancellation (35)	7 (10)	19.5 (22.5)	.018^a^
Overlapping figure (20)	6.5 (6.3)	16.5 (5)	.002^a^
Navon figure (12)	5.5 (7.3)	12 (2)	.001^a^
Visual-perceptual			
Matching test (10)	7 (3)	10 (2.75)	.004^a^
Cookie theft picture (15)	7.5 (6.5)	9.5 (4)	.044^a^

Data are shown as median (IQR) or mean (SD). Numbers in parentheses next to the test name are maximum possible scores. MMSE, Mini-Mental State Examination; MoCA, Montreal Cognitive Assessment; CDR-SOB, Clinical Dementia Rating-Sum of Box; AVLT, Auditory Verbal Learning Test

^a^Kruskal-Wallis one-way ANOVA followed by post-hoc test

^b^ANOVA followed by Bonferroni test

**Fig. 2:**
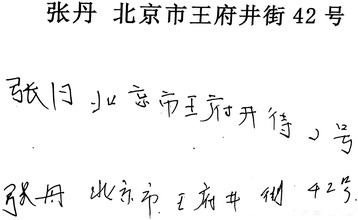
Examples of handwriting from two PCA patients. Chinese characters are abnormally formed with stroke omissions, word substitution, and unintelligible words. The first row is the sample to be copied; the second row is the copy produced by Patient 1; and the third row is the copy produced by Patient 15

Language abilities differed between PCA and EOAD patients. The ability of PCA patients to write was significantly more impaired than in EOAD patients (*p* = .002). PCA patients showed pronounced incomplete words (*n* = 12), word substitution (*n* = 7), unintelligible characters (*n* = 11), character structure impairment (*n* = 16), picture drawing (*n* = 5) with respect to writing Chinese characters. For example, if the direction of stroke was not horizontal or vertical, the stroke was not completed, the enclosed structure was not closed, or a compound character was separated into two or three parts, the character was considered structurally impaired (Fig. 2). Some scripts are appeared scribbled (*n* = 16) and worse than previous scripts. PCA patients showed significantly more impaired reading ability than EOAD patients (*p* = .002), manifesting as missed words (*n* = 17), skipped lines (*n* = 14), misreading (*n* = 10), repeated reads (*n* = 4), and failure to recognize Chinese characters (*n* = 7). Moreover, PCA patients performed worse than EOAD patients in the bell cancellation task (*p* = .018), overlapping figures (*p* = .002), Navon figures (*p* = .001), and matching test (*p* = .004), as well as the Cookie Theft picture (*p* = .044). There were no significant differences between PCA and EOAD patients in AVLT, digit span, trail making, BNT, verbal fluency, calculation, MMSE, MOCA, or CDR scores.

## Discussion

We compared the cognitive manifestations between PCA and EOAD in Chinese patients. All patients had disease durations under 4 years. The results showed PCA patients to have greater impairment on visuospatial and visuoperceptual tests than EOAD patients, while PCA patients were significantly impaired in Rey-Osterreith complex figure copying, writing, and reading tests, with symptoms of visuo-constructive agnosia, alexia, and agraphia. However, both PCA and EOAD patients had impairments of calculation, episodic memory, working memory, executive function, picture naming, and verbal fluency in the present study. The difference in PCA and EOAD in cognitive features could help the differentiation in clinical diagnosis during early stages of the disease.

PCA patients had more severe symptoms visuospatial and visuoperceptual symptoms than EOAD patients. These results are consistent with earlier reports [2, 11, 29]. Visuospatial and visuoperceptual dysfunction arises from dysfunction of the dorsal streams (occipito-parietal pathway) and the ventral streams (occipito-temporal pathway) [30]. These higher visual dysfunctions are core clinical and cognitive features that define the PCA syndrome. However, PCA lacks standard neuropsychology test profiles for diagnosis or further clinical trials. These cognitive tests evaluating visual function may become a very quick-acting tool that could be used to distinguish PCA from EOAD.

We found that a significantly higher proportion of PCA patients scored lower than EOAD patients in figure copying tests. Figure copying is mainly related to visuo-constructive function, spatial processing, and visual neglect in bilateral parietal occipital lobe function [31, 32]. The figure copying deficit in our patients may be associated with dorsal stream lesioning [30]. Our findings confirm the results obtained in a previous study [9], which revealed that visuo-constructive impairment is more prominent in PCA patients than in EOAD patients. Our results also indicate that no patients with PCA had normal scores in Rey-Osterreith complex figure copying task, indicating the visuo-constructive function distortion. This test of Rey-Osterreith complex figure copying has been reported to have a 100% sensitivity and specificity with respect to distinguishing PCA from typical AD [9].

PCA patients were more impaired in writing tests than EOAD patients, suggesting that agraphia is an important cognitive feature of PCA. The impairment manifested in the form of incomplete word (stroke omissions and iteration), word substitution, picture drawing and changes in calligraphy [33, 34], in both Euro-American and Chinese PCA patients. These errors might suggest limb apraxia or visual disorientation [34, 35] usually associated with damage in the left angular gyrus and nearby regions in the parietal and occipital lobes [36]. However, in our study, another prominent presentation on writing disorders is characterized by unintelligible characters (61%) and structurally impaired characters (89%) in Chinese patients, presumably due to impairment in visuo-constructive functioning. The Chinese writing system has some unique features: It uses square-shaped characters, and it differs from alphabetic writing systems in the visual features of its orthography. Chinese characters are commonly referred to as logographs and involve combination and construction [37]. It suggests that Chinese scripts would reflect visuo-constructional ability more sensitively than alphabetic scripts, possibly accounting for PCA disrupting more visually complex logographic writing systems than alphabetic system [35]. The visuo-constructional impairment on writing test of Chinese PCA patients would be a good identification tag for PCA. A cross-cultural study recently reported that alphabetic or syllabic writing systems exhibit a static pattern, not changing over time over the last three millennia [38]. If logographic writing systems follow a similarly static pattern, we might be able to develop the writing test of Chinese characters into a sensitive diagnostic neuropsychological tool usable by individuals who do not speak or read Chinese. Because writing impairments are nonvisual disorders, clinicians might dismiss them as patient complaints. Testing the writing abilities of patients may uncover evidence of subtle impairments in visual cognition. Moreover, the writing error profile in Chinese patients was unique and may facilitate the diagnosis of PCA.

Reading ability was far more impaired in PCA patients than in EOAD patients, suggesting that alexia is a neuropsychological marker suitable for differentiating PCA from EOAD. This symptom appears often (90%) and early in PCA patients. In our study, PCA patients presented with missing words (94%), getting lost on the page (86%), and getting lost from one line to the next (67%). These clinical complaints in PCA have been attributed to visual disorientation [2, 24]. Some PCA patients misread similar characters or pronounced only half or part of the character, which may be due to visual neglect, especially considering the compound structure of many Chinese characters. Our PCA patients also exhibited another type of alexia: visual alexia (failure to recognize Chinese characters), also called pure alexia. This is thought to result from the destruction of the visual word form system or from deprivation of visual input. Visual form processing as part of the visual “what” pathway can be identified letters or graphemes [39, 40]. The visual word form can then trigger retrieval of the character’s meaning, grammatical features, pronunciation, and other characteristics. Again, English and Chinese writing systems are remarkably different: First, structurally, a Chinese character is composed of strokes and components, which is different from English, which is built on single alphabetic characters. Second, the process of reading Chinese characters involves orthographic and phonological conversion, but reading English involves morphemes and phonetic conversion [41]. Thus, in an alphabetic system, patients with pure alexia often use a style of compensatory reading known as letter-by-letter reading [42], but this does not work in Chinese system. The visual dysfunction disrupted the conversion of the visual word form to the orthographic, which may leads to pure alexia in Chinese PCA patients [43]. In the future, we plan to investigate the relationship between the visual form processing of Chinese characters and the ability in PCA patients to read as an indicator for pathogenesis of alexia.

We found episodic memory impairment in both PCA and EOAD patients during the early stages of the disease, as indicated by AVLT, but the two conditions did not differ from one another. Memory impairment may therefore be expected to yield poor results when used to distinguish PCA from EOAD. Our results are consistent with those of Charles and Hillis [9] and Ahmed et al. [44], who found memory to be impaired in both PCA and typical AD patients. Kas et al. [11], however, found that PCA patients performed better than typical AD patients on an episodic memory test, even during the early stages of PCA (≤ 3 years since onset). Additionally, the diagnostic criteria for PCA suggest that episodic memory is relatively preserved. This discrepancy in the research literature may be due to different durations of disease, different sample sizes, different memory tests, or some combination of those elements. Because reliability of results depends on the accuracy of diagnosis, our entire sample of PCA patients underwent imaging (FDG PET or SPECT) to ensure sufficient accuracy. In PET amyloid imaging, the majority of our PCA patients had AD pathology with elevated beta-amyloid deposition and diffusion in the cortex. These common pathological bases suggest a reason that both PCA and AD have memory impairment [44].

Despite the evident contrasts between PCA and EOAD patients in the present research, we urge caution in the interpretation of our results. The sample sizes were relatively small, which was unavoidable, owing to the relative rarity of PCA and the difficulties in recruiting participants during the early stage of symptom onset. As result of the small samples involved, it is possible that true differences exist between the PCA and EOAD groups, but that we did not find such differences because the small sample sizes give the experiment limited statistical power. However, we also emphasize that, along with meeting clinical criteria, neuroimaging verified that all our PCA patients had been accurately diagnosed. Most of the PCA patients also underwent PIB-PET to confirm diagnosis by AD pathology. Although nine PCA patients lacked pathological results, for minimize confounding and bias, we followed up those patients for at least 12 months to support AD diagnosis by excluding Lewy body disease, corticobasal degeneration, or prion disease. The PCA and EOAD groups were also matched for disease duration, which may have minimized the clinical differences between PCA and EOAD. Another constraint of our study is neuropsychology measures. Some neuropsychology measures (e.g., SSST, reading and writing tests) were brief and quick for practitioner, but they may result in underestimation of the extent of impairment, and result in ceiling effects in our data (e.g., a bunching of scores at the upper level in control group). Ceiling effects would also tend to obscure true differences between groups. In the future, our studies will systematically examine participants using neuropsychological tests on calculation, reading ability, and writing ability in PCA.

## Conclusion

PCA patients present significant cognitive impairments including visuospatial, visuoperceptual, visuo-constructive, writing, and reading. As a diagnostic battery, those tests could be used to reliably distinguish PCA from EOAD. Tests of handwriting and reading appear to have a particularly high specificity for identifying PCA in Chinese-speaking patients. It would be practical to incorporate this battery of tests into existing cognitive screening for PCA, and it could be used to establish a brief and routine PCA neuropsychological examination for improving the early diagnosis of PCA.

## Abbreviations

AD: Alzheimer’s disease
ADL: Activities of daily living
AVLT: Auditory verbal learning test
BNT: Boston Naming Test
CDR: Clinical dementia rating
CT: Computed tomography
EOAD: Early-onset Alzheimer’s disease
FDG: ^18^F- fluoro-deoxyglucose
IWG: International Working Group
MMSE: Mini-Mental State Examination
MoCA: Montreal Cognitive Assessment
MRI: Magnetic resonance imaging
PCA: Posterior cortical atrophy
PET: Positron emission tomography
PIB: [^11^C]-labeled Pittsburgh compound B
SPECT: Single photon emission computed tomography
SSST: Series of Seven Subtraction Test
